# Refugee Workers' Agency: Displaced Persons Shaping Relief and Rehabilitation Work in Postwar Austria and Germany

**DOI:** 10.1177/00220094251396883

**Published:** 2026-03-23

**Authors:** Franziska M. Lamp-Miechowiecki

**Affiliations:** 27258 University of Vienna, Austria

**Keywords:** Central Europe, postwar, humanitarianism, United Nations, displaced persons, refugees

## Abstract

At the end of the Second World War, Allied-occupied Austria and Germany became places of transit for hundreds of thousands of displaced persons (DPs) and refugees, as well as sites of employment for various internationally composed teams of relief workers. Through its analysis of personal documents produced by both United Nations Relief and Rehabilitation Administration (UNRRA) and Quaker relief workers, administrative correspondence by UNRRA and later the International Refugee Organization (IRO), organizational bulletins, as well as the diary of a DP, this paper shows how DPs themselves played an active part in the postwar rehabilitation of displaced people. Their duties within the camps and their close collaboration with non-DP relief workers highlight various forms of refugee agency. This paper further examines what UNRRA and the IRO gained through their collaboration with DPs, as well as to what extent DPs could themselves benefit from this. Despite the hierarchies embedded in the labour policies of UNRRA, the IRO and the Allied occupation forces, this paper demonstrates that focusing on refugee workers and the diverse ways in which they utilized their skills to administer relief measures and sustain camp life reveals the interrelation between refugee agency and the agency of non-DP relief workers.

With reference to the postwar Displaced Persons (DPs) camps, Gemie et al. noted that ‘at least three forms of authority interacted’, namely that of the occupying forces, the United Nations Relief and Rehabilitation Administration (UNRRA), ‘and lastly, often unrecognized, the DPs themselves’ who ‘developed their own forms of authority’.^1^ What Gemie et al. call ‘authority’, this paper would like to conceptualize as different forms of ‘agency’. The main goal of this article is to analyse examples of the DPs’ own ‘forms of authority’, with a special focus on those DPs who were engaged in various roles within the camps, such as camp leaders, police chiefs or translators, or who were active within the camp administration or employed by UNRRA. In the following pages, these individuals will be referred to as ‘refugee workers’^2^ . This term is used here in a broad sense and includes both workers who received monetary compensation for their work as well as those who worked to receive certain goods or benefits for their lives in the camps. This paper does not examine work done by DPs outside of camp structures – for example, for military authorities or German industry – nor their involvement in educational or vocational training programmes, as these topics have been more thoroughly explored in other studies.^3^ While also not the subject of this paper, the important role of refugee workers for postwar reconstruction requires further study.^4^

Rather than viewing the agency of those providing care as separate from that of those needing care and support, this paper seeks to examine phenomena which demonstrate the connectedness of the experiences of the international – mostly American and British^5^ – staff working for UNRRA, or voluntary organizations like the Quaker Friends Relief Service, and refugee workers. While previous research has mainly focused on the labour requirements for DPs and elements of coercion present in the Allied labour policies regarding DPs,^6^ this article would like to look at the topic of DP labour from another perspective, namely that of agency. It understands agency, broadly defined, as ‘the role of the individual as an active force in constructing his or her life course through the choices and actions taken’.^7^ It considers whether and in what ways the actions of DPs working for UNRRA or in various positions in the camp resulted in different forms of ‘personal’ and ‘proxy agency’.^8^ Personal agency, as understood in this paper, comprises actions taken by DPs themselves for their personal benefit. Proxy agency, on the other hand, refers to ‘the capacity of an individual or group to act for another individual or group in specific social transactions’.^9^ It must be emphasized that personal agency, as understood here, cannot be analysed in isolation from other forms of agency, such as proxy agency, since acting on one's own behalf (personal agency) inherently includes the potential to act on behalf of others (proxy agency), and vice versa.

Another key argument pursued in this paper is that through the employment of DPs in the relief and rehabilitation work, the agency of DPs and non-DP relief workers was overlapping and interrelated. As this paper will show, the agency of DP workers, which becomes evident through their important roles and duties inside the camps and their active involvement in the provision of care and assistance, greatly benefited the organization's mandate; indeed, the administration of postwar relief work would not have been possible without the active engagement and skills of DPs themselves. It also illustrates how their engagement with the organizations responsible for providing relief and rehabilitation could, in turn, influence the DPs’ own agency. An UNRRA document on collaboration with DPs in the management of refugees highlights the important role of the work done by DPs:

There is no doubt that there are many persons in the Displaced Persons Camps throughout Germany and Austria who can easily do the work of Class I employees in camps. Actually, if the facts were known, the bulk of the work even now is being done by DPs under the supervision of Class I employees.^10^

Bearing in mind this close collaboration of DPs with non-DP relief workers, referred to in the quote as Class I employees of UNRRA, this paper therefore suggests adding the concept of interrelated agency to that of personal and proxy agency.

In preparing this paper, relief workers’ reports and institutional correspondence were closely examined and read against the grain to locate descriptions of refugee agency and interrelated agency between DP and non-DP camp staff. One of the questions explored here was what these texts can reveal about the agency of individuals other than the narrators. Sources created by relief workers in fact repeatedly mention the agency of DPs, as well as their reliance on the assistance of DPs for carrying out postwar relief work among Europe's displaced populations. However, research on relief work in postwar Europe has not gone so far as to examine their reports and memoirs from this perspective. In this context, this paper asks what information personal narratives by non-DP American and British relief workers can provide about refugee agency and how they should be treated as sources that contain power hierarchies. The aim here is to move beyond ‘the conceptualization of DPs as recipients’ which had functioned as ‘a necessary corollary to UNRRA officers’ self-perception (and self-depiction) as rescuers’,^11^ as Silvia Salvatici points out. Salvatici shows how UNRRA's official policies, as well as ego documents created by relief officers, often focus on the vulnerability of DPs.^12^ This article, however, seeks to examine these sources through a different lens, highlighting that they also contain accounts of refugee agency.

While acknowledging the fact that the descriptions of DPs by relief personnel were often characterized by paternalism and the use of stereotypes, as Salvatici and Aksamit have accurately observed,^13^ the memoirs, diaries and reports by aid workers examined in this study also demonstrate that the relationship between relief personnel and those they set out to care for was far more complex than being strictly hierarchical. In fact, the international relief staff worked together with DPs and relied on their help and expertise. Furthermore, the functions performed by DP workers and representatives – for example, doctors, nurses, camp leaders, police chiefs and translators – and their important role in managing camp life could create new opportunities for empowerment and agency among the displaced populations.

The analysis of relief workers’ reports for descriptions of refugee agency and examples of fruitful cooperation with DPs demands a brief examination of the context in which the sources for this paper were created. One of these is ‘a socio-psychological study of Displaced Persons in UNRRA camps’^14^ by Audrey Duchesne-Cripps, which focuses on the mental state of DPs of different nationalities dispersed in various camps of the British zone in Germany, where Duchesne-Cripps worked as a UNRRA welfare officer until June 1947.^15^ In the unpublished diary of UNRRA worker Thomas S. Hall, another key source for this paper,^16^ the reader is mostly confronted with descriptions of the training of UNRRA personnel, the daily life in DP camps in Austria and Hall's interactions with refugees, as well as his conflicts with military personnel. Hall's diary is a fascinating historical source, as it reveals not only how he saw himself as an UNRRA employee but also how and to what extent he interacted with DPs. It therefore also reveals a lot about how he, as a relief worker, viewed the DPs’ roles within the camps and what agency he attributed to them.

Hall's description of his postwar experiences in Europe is, however, far from the only account by relief workers in the field.^17^ Another example of this is the book *By the Rivers of Babylon* by Margaret McNeill, which mainly focuses on her time with Polish DPs and was published in 1950. McNeill worked as a relief worker for the Quaker Organization Friends Relief Service and was part of one of 14 Quaker teams caring for DPs in Germany.^18^ Another important source for this paper is the autobiographical book *The Wild Place*^19^ by the American UNRRA worker Kathryn Hulme, which mostly concentrates on her work and experiences in the Wildflecken DP camp in Germany.

When focusing on agency in refugee contexts, one has to ask who can be considered to ‘have access to resources or expertise or’ to ‘wield influence and power’.^20^ Of course, those who had the authority and who stood at the top of the ‘Allied relief regimes’^21^ in the camps were the Allied military authorities, as well as aid workers representing internationally operating relief organizations like UNRRA.^22^ They provided the financial means and material goods and laid down the rules for camp life.

Despite these hierarchies, however, DPs were often able to exercise a certain degree of agency. How and where this agency emerged is a central concern of this paper, which focuses on the DPs’ work inside the camps in general as well as for UNRRA and the International Refugee Organization (IRO) in particular. During the mandate of UNRRA and later IRO, DPs were working in the camps in private workshops or were active as camp or national leaders, while others were employed by UNRRA or the IRO as so-called ‘Class II’ personnel. In all these roles, DPs found themselves in an in-between position of both receiving and providing care and relief, from which different forms of agency could arise.

In the British and French occupation zones, those DPs who could work had to do so to receive care and maintenance,^23^ although ‘regulations stipulating requirements for compulsory employment were only approved in January 1947 in the British zone’.^24^ In the French zone of occupation in particular, there were very strict rules regarding the employment of DPs. As Historian Laure Humbert points out, ‘UNRRA officials condoned French policy of work and justified it by evoking the “therapeutic” and emancipatory virtues of work’.^25^ In the American zone of occupation, on the other hand, ‘forced labour for refugees was not official policy’.^26^ UNRRA's regulations with regard to compulsory labour among DPs were rather ambiguous and varied across different groups of DPs. Although UNRRA encouraged DPs to work, certain exceptions were made regarding persecuted groups of DPs, like Jewish survivors.^27^ Silvia Salvatici points out the ambiguities of the IROs and the Americans’ labour policies and the experiences of DPs: ‘Although formally IRO and American policy did not make work compulsory, DPs seemed to perceive it as if it did’.^28^

While Blumenthal points out that DPs ‘welcomed the opportunity to keep themselves occupied with meaningful, productive work’ inside the camp, she also emphasizes that for UNRRA, the employment of DPs in different positions in the camps was ‘both a means of control and a form of welfare’.^29^ Blumenthal argues that the goal of the organization's policy towards DP labour was ‘to inculcate disciplined, high-quality work and honest shop conduct among the workers’.^30^ Silvia Salvatici's article takes a similar approach, analysing the work performed by DPs for military authorities and UNRRA more through the lens of ‘direct and indirect compulsion’^31^ rather than emphasizing agency. Although the hierarchies and elements of coercion that characterized DP labour in the refugee camps cannot be ignored, this paper takes a different approach by asking firstly what the DPs could gain from working in the camps and for UNRRA or the IRO and secondly what UNRRA and the IRO could gain through their employment.

Contemporary observers have described DP camps as ‘immense pools of manpower representing every known skill’.^32^ And, indeed, in the refugee camps, every profession and every trade was to be found among the inhabitants. Those who had not yet had the opportunity to learn a skill could do so now. The training of DPs for job placements was one of the main goals of UNRRA, besides facilitating the DPs’ repatriation: ‘Throughout 1946 […] the aim and policy of UNRRA in the British zone has been directed towards two ends: repatriation and employment of the displaced persons under its care’.^33^ The occupational training of DPs was also directly connected to the use of their workforce by military and civilian authorities or UNRRA.^34^ Furthermore – and especially so under the IRO's mandate – vocational training was intended to enable DPs to gain knowledge and expertise for their future life in the West.^35^ UNRRA argued that by training and employing DPs, it created new possibilities for agency among the displaced people. In February 1947, for example, the *UNRRA Team News* proudly proclaimed ‘the enthusiasm of the DPs to learn new trades or to take refresher courses’, which would ‘equip them for the future’.^36^

While it is important to recognize that the labour of DPs was actively sought and encouraged by both the Allies and organizations such as UNRRA and the IRO, this paper demonstrates that its significance extends beyond this. It aims to demonstrate that examining DP labour offers valuable insights into the lived experiences and agency of DPs themselves. The following section of this paper specifically examines the personal and proxy agency that could emerge from DP labour for DPs themselves. It places particular emphasis on the labour of DP women – a topic that has been largely overlooked in existing research^37^ – as well as on the agency of DPs working as UNRRA staff, translators, police chiefs, or doctors. The goal is to explore the broader implications of labour on refugee agency.

One of the effects that being employed by UNRRA or working inside the camp administration, in workshops or as camp leaders, had for DPs themselves was a potential improvement in their displacement experience and living conditions. For some DPs, the opportunity to learn new skills, work in their profession and gain employment inside the camp administration could make the displacement experience easier to endure.^38^ As Margarete Myers Feinstein argues for female Jewish refugees, ‘their participation in the DP economy […] revived their feelings of self-worth and sustained their families’.^39^ With regard to the DP camp in Bad Gastein, Blumenthal argues that the DPs’ engagement in ‘workshops and camp maintenance formed part of the survivors’ attempts to regain control of their lives’^40^ and their own agency. Feinstein points to another interesting dimension of refugee agency when she writes that the creation of ‘seamstress shops, restaurants, and other businesses […] enabled Jewish DPs to minimize their contact with Germans’.^41^ So, running their own businesses allowed the DP community to be more independent from local suppliers.

Refugees were far from being mere recipients of aid; they played an active role in managing the camps, producing various goods in workshops and serving as teachers and nurses for their fellow DPs. Despite this fact, the pivotal role that DP women in particular played in postwar rehabilitation was not sufficiently acknowledged in accounts by UNRRA, as Feinstein points out.^42^ In her work, she highlights their important role for the DP community. She describes how women with specific skills found work in various areas, such as assisting the military, opening private businesses within the DP community or taking teaching roles in schools organized by the Organization for Rehabilitation and Training. These activities played a significant role not only in helping the women regain a sense of purpose and self-worth but also in supporting the recovery of the DP community as a whole. The training and employment of DPs as nurses and doctors, for example, not only helped to meet the demand for medical personnel^43^ but also reduced the community's ‘reliance on German doctors and nurses’, which had ‘certainly created tensions’.^44^

The refugee agency also becomes visible in protests and in the demands put forward by DP workers. Feinstein demonstrates in her research on Jewish women that DPs should not be regarded as ‘passive workers’, rather ‘they sought to organize and to press their demands for better treatment’.^45^ She mentions an example of this from Camp Feldafing, in Bavaria, where ‘the working women in the offices of the Camp Administration’^46^ demanded to receive shoes for their services. In her memoir, Hulme also mentioned a protest by DP workers. When new food parcels arrived in the camp, the DP woodworkers demanded that they be distributed at once and not in smaller portions over a longer period of time: ‘Our DP workers staged a sit-down strike. Eight hundred woodcutters who went daily to the woods refused to get into the waiting trucks’.^47^ These examples show that refugee workers were able to organize themselves and make their voices heard, which, because of the importance of their work for the camp economy and infrastructure, could have a direct impact on the entire camp population.

The question of compensation for the work done by DPs is, of course, also highly relevant for assessing the agency they could gain through their employment. As Blumenthal shows for the DP camp in Bad Gastein, DPs employed in various private workshops in the camps were paid through a points system, which allowed DPs to purchase ‘food, toiletries, cigarettes, clothing or shoes’, or by receiving ‘larger food rations’^48^, rather than in wages because ‘the organization viewed any activity that generated cash as black-market profiteering and prohibited it’.^49^ With regard to the work done by DP women in the camps, Feinstein also mentions that it was very poorly paid in monetary terms, if at all. Often, they were only given certain products in exchange for their work, or additional rations which, however, they could exchange to buy something on the black market.^50^

To a certain extent, using one's skills for professional purposes in the camps could improve the living circumstances of DPs and guarantee a certain economic independence, as Feinstein points out by using the example of Hilde Mantelmacher, a Jewish DP, who worked as a seamstress:

I was knitting for the American soldiers. I made scarves and I made hats and I made masks. I started to work because I got extra food if I worked. I got candy bars. […] I knitted all the time in Landsberg [DP camp]. […] So I had boots that they [other camp inhabitants] made me for my candy bars.^51^

Feinstein emphasizes that ‘this trade economy created opportunities for those entrepreneurs […] who could establish […] businesses’^52^ and thereby recreate a certain sense of normal life.

Besides improving their living conditions in the camps and rebuilding their communities, assuming various official roles within the DP camp system could further enhance the DPs’ personal agency. In her memoir on her time in Camp Wildflecken, Hulme, for example, mentioned an interesting dimension of refugee agency with regard to a DP police chief in the camp. She remembered how he had uncovered the black-market activities of ‘two German women whom he had caught trafficking in cigarettes in the camp’. Instead of simply ‘turning the arrested women over to the proper outside authorities’, he decided to ‘set them to scrubbing the floors of his police headquarters, meanwhile inviting in the camp inhabitants to have a good long look at their former conquerors down on their knees beside a scrub bucket’.^53^ The position as DP police chief seemed to have given him the authority to carry out such an act of punishment and retribution.

In addition, research for this paper has shown that employment with UNRRA's successor, the IRO – which focused more on emigration and resettlement as a means of rehabilitation for DPs – could further increase the agency of DPs by enhancing their emigration prospects. Hulme illustrated how DPs employed by the IRO in resettlement centres, for instance, had access to information about the emigration process that other camp residents may not have had. In her memoir, she demonstrated how working for the IRO could enable DPs to gain valuable insights into the whole emigration apparatus. She told the story of Stanislawa, a Polish DP who had survived the concentration camps and ended up as a translator for the director of an IRO resettlement centre:

Stanislawa had leapt at the opportunity of employment in the Resettlement Center because it brought her closer to the sources of power. She had studied the idiosyncrasies of the mission men […] and had already observed that late afternoon […] was the most favorable time to have an emigration interview, that well-groomed applicants got through the initial encounter faster than the slovenly ones.^54^

Her role as an IRO translator enabled Stanislawa to learn a great deal about the management of resettlement. By working so close to the resettlement missions, she gained knowledge about how best to navigate the emigration system. Hulme recalled her saying to her, ‘all this I make for reconnaissance only, for that great day when your country will start to call forward. It is well to know […] the ropes’.^55^ Although this episode once again illustrates the agency of refugees through the perspective of a non-DP aid worker, it also alludes to the important fact that working alongside international staff and representatives of resettlement missions could help refugee workers in their own attempts at resettlement and thereby increase their personal agency.

Furthermore, a close reading of the relief workers’ memoirs showed that working as part of the DP medical personnel in an IRO resettlement centre could also create interesting possibilities for proxy agency. In her memoir, Hulme gave examples of this kind of agency exercised by DP doctors working in the resettlement medical centres:

The DP doctors had themselves arranged that no one of them would check a countryman, but that the Latvian doctor would examine the Lithuanian DPs, the Lithuanian doctor the Ukrainians, the Polish doctor the Latvians and so on, thus protecting their medical ethics from the fierce temptation to push a countryman through if they could.^56^

This quote makes apparent the influence that DP doctors could – but in this case apparently chose not to – exert on the results of the eligibility screening for resettlement. It is very interesting to see here that for the DP doctors, it seemed to have been important to be considered ‘neutral’ and ‘objective’ towards their own nationals. However, this did not prevent them from acting on behalf of their fellow DPs at a later point. In her narrative, Hulme goes on to describe how DPs were rejected for resettlement due to their high blood pressure:

Our DP camp doctors who had examined them [the DPs, who were screened for resettlement] just before call-forward swore that there must be something wrong with the blood pressure apparatus at the Resettlement Center. […] We [the UNRRA relief workers] were not taken into their confidence, but one day they told us there would be no more deferrals for high blood pressure […]. Long afterwards we learned that they […] were quietly injecting their excitable called-forward countrymen with something like scopolamine [a sedative] and sending them forward to the frenzy of the Resettlement Center in a gentle `twilight sleep´ which kept the blood pressure from soaring insanely until after the Center medical check was made.^57^

This episode demonstrates how DP doctors bypassed the authorities of the camp and quietly set out to accelerate the resettlement process by injecting the DPs with some kind of sedative to decrease their blood pressure so that they would not be considered ineligible for emigration. This example shows that DP doctors could apply their medical knowledge in order to aid other DPs during the immensely stressful resettlement process.

Hulme also mentions similar instances of proxy agency by DPs, such as when she and her Polish translator – known as ‘the Countess’ – were issuing provisional passports to DPs:

The Countess was a tall handsome woman in her early sixties. […] I quickly learned to know in advance, by watching her face, something of the shape of the problem being unfolded to her in halting Polish, Russian, German or Ukrainian before she recited it back to me in flawless Warsaw-society French.^58^

The UNRRA worker's reliance on her language skills allowed the DP translator to act on behalf of other DPs, as becomes clear in two passages in Hulme's narrative. On one occasion, Hulme and the Countess encountered a Polish botany professor who requested permission to leave the camp to search for certain plant species in the forest. At first, Hulme did not want to fulfil his wish, because ‘the woods were off limits’ for DPs. However, then the Polish Countess stepped in and advocated for granting his demand:

‘It's all he has left in the world’, the Countess pleaded. ‘His entire family killed […] now he has only his flowers’. Her eyes forced me to pick up my rubber stamp, the seal of Wildflecken which could transform any bit of paper into a passport. On the blank line after ‘Destination’ I wrote in ‘Botany’ and prayed that the GI guard at the main gate was illiterate.^59^

This quote shows quite clearly how much influence DP co-workers could exercise on the authorities in charge – most of the time non-DP relief workers. Another similar episode in Hulme's narrative shows how the Polish Countess acted as a mediator between DPs and non-DP relief workers. She recounted how one day the distribution of food parcels was coordinated by a group of displaced children. When they started to sort the packages, Hulme was surprised by how well they were ‘stacking their individual products in automatic patterns of neatness and efficiency’. After some time, she learned that these children and young adults had worked as forced labourers in German factories. As soon as she knew what was going on and why they knew how to handle the products so well, Hulme wanted to dismiss them from this job. However, here again, the Polish Countess intervened and argued:

‘Oh, but you cannot,’ […]. ‘You must believe me, they are happy here. If you could only understand what they are saying to each other. This is something new and exciting to do. Their pride […] if you dismissed them now, they would feel they had failed you’.^60^

And indeed, Hulme, later on, when she wanted to employ other DPs to do this task, observed that the young DP girls and boys were very unhappy with this decision.^61^ Because the Polish Countess could understand what the boys and girls were saying, she could act on their behalf – showing once again the importance of language skills for understanding DPs and for exercising proxy agency.

After having examined several examples of the personal and proxy agency among DP workers – some of them employed by UNRRA, others working in various roles inside the camp – this paper would like to discuss one last example of these forms of agency among refugees by highlighting the role of DP leaders. As camp and national leaders, DPs acted as intermediaries between relief workers, military authorities and other DPs: ‘DPs were recruited from the start to co-run their own affairs, and “national leaders” were appointed or elected to represent their cohort’.^62^ The DP camp leaders played a key role in the ‘self-organization and self-representation’^63^ of DPs, a practice actively supported by UNRRA, the IRO and the Western Allies – particularly the United States.^64^ Relief workers’ memoirs report that DP leaders were elected by the DPs themselves, as an example from Hall's diary shows: ‘To-day [sic] at 20.30 will be a meeting of all Latvian [sic] in the Theatre to elect another leader’.^65^ Duchesne-Cripps also wrote that the camp leaders ‘would be chosen by the [displaced] people themselves’, which makes them an ideal example of refugee proxy agents. She mentioned that ‘the principles of democracy, especially in relation to elections, were normally insisted on in the choosing of a camp leader’.^66^ The representative function of DP leaders was also hinted at by the historian G. Daniel Cohen, who emphasized their intermediary position by stating that ‘elected leaders formed governing committees who represented the DPs in their dealings with civilian and military authorities’.^67^

What becomes evident in relief officers’ testimonies is that there was an active exchange happening between non-DP relief personnel and DP leaders. In his diary, Hall mentioned multiple times talks with the ‘leaders’ of DPs (‘Roumanian [sic] leader instructed regarding movement of his nationals’ or ‘Weekly meeting of all camp leaders and special meeting of Committee of National leaders’ and ‘Domestic matter discussed with Yougoslavian [sic] leader’^68^). McNeill also recounted an interesting episode regarding the proxy agency of DP leaders when she described that she ‘met the representatives of the three Baltic nations’. McNeill remembered how, during the meeting, the DP leaders of Lithuania, Latvia and Estonia advocated for an improvement of their fellow nationals’ living circumstances. The representative of Lithuania in this context emphasized the agency of his nationals as well as their skills and work ethic: ‘Give me any kind of building, even if it is in bad condition […] and my people will themselves get the camp into order’.^69^

However, as Katarzyna Nowak has shown, DP camp leaders did not always have their fellow DPs’ interests at heart but often followed an ideological agenda. She describes how Polish national leaders had a lot of influence on the daily life in the camps: ‘medical reports from United Nations Archives (New York) show how UNRRA workers and Polish leaders regulated intimate life and matters of public health in the DP camps’.^70^ She further mentions the important role of DP leaders and what she calls ‘elites in the DP camp’ for ‘national and religious revival’.^71^ Examples from the diary of the DP Anton Žakelj also illustrate that serving as a refugee leader could lead to the misuse of authority. In July 1945, he noted in his diary that the refugees who were responsible for the management of the camp under British direction were reluctant to relinquish their authority. In a diary entry in November of the same year, Žakelj describes how – also in direct opposition to UNRRA regulations – some DP leaders used their role to their own advantage by using their position to skip the line during the distribution of food and by taking more food than had been assigned to them.^72^ These examples show how the prominent position of these leaders within the camp could amplify their personal agency and, in some cases, could also result in abuses of power.

While the previous section of this paper primarily explored the personal and proxy agency that could arise from the work of DPs in various occupations and positions within the camps, the following section will shift its focus to the impact that working directly with UNRRA or the IRO as part of the organizations’ local staff could have on the DPs’ experiences and agency, as well as on those of the relief authorities.

DPs officially recruited by UNRRA were primarily employed as so-called Class II personnel.^73^ These employees received wages, which were, however, ‘paid in local currency’. Often, DPs who worked for UNRRA were not paid by the organization itself but ‘by the local Burgomaster [sic] [meaning Mayor] in Reichsmarks at the established rate of wages for the area’.^74^ The majority of the 1893 Class II workers employed in March 1947 in the care and administration of DPs in Germany were of Polish, Latvian, Estonian, French or Lithuanian nationality. A high percentage of them (15.6 per cent) were considered stateless.^75^ Although in her study, Duchesne-Cripps did not fail to mention the precarity of these UNRRA Class II employees and the unregulated sphere in which they operated, she also pointed out that there were certain benefits for working as part of this organization. For example, DP staff had access to army rations, cigarettes, and a uniform,^76^ which resembled that of the UNRRA's international personnel.

Despite UNRRA being a very important employer in the postwar period,^77^ insufficient attention has been paid to this part of the organization's history. In particular, research has so far failed to acknowledge the important roles played by DP Class II employees in the implementation of the organization's relief and rehabilitation policies. This paper reveals that through the employment of DP staff, UNRRA could delegate important tasks to refugee workers, who were thereby improving the functioning of the organization. The DPs’ work and collaboration with UNRRA contributed to advancing and fulfilling the organization's mandate and, at the same time, to increasing the agency of non-DP relief workers. The organization's ability to fulfil its mandate was closely tied to its collaboration with DPs and can serve as an example for the concept of interrelated agency, which was developed as part of this paper.

To better determine whether working for UNRRA or the IRO as Class II employees created new opportunities for agency among the DPs themselves, the motivation behind the organization's decision to employ DPs needs to be ascertained. Although rehabilitative and humanitarian considerations certainly played a role here, it can be argued that UNRRA's rationale was mainly dominated by economic considerations, as Class II employees were significantly cheaper per person than Class I employees^78^ as well as by other diverse advantages that the organization saw in the employment of DPs – such as their language skills and the closeness of their own displacement experience to that of their fellow DPs. As Salvatici has described, ‘DPs wearing UNRRA flashes often proved to have skills that the “rescuers” didn’t have, skills essential for the administration of the assembly centres’.^79^ The use of DPs as part of its staff was a deliberate policy by UNRRA, which tried to ‘work more and more through the Displaced Persons themselves’.^80^ This aligned with the organization's motto for establishing vocational training programmes to ‘help the people to help themselves’.^81^ Looking at the organization's employment policies, we can see how crucial refugee workers were to its mandate, and how power relations within the organization, and the question of who was to provide postwar relief, need to be re-examined in this regard.

Most of the time, sources by relief workers do not explicitly state whether individual refugees acting in official functions within the camp were employed by UNRRA – for example, as Class II employees. In Austria and Germany, DPs could be ‘employed in camp operations, self-employed in camp workshops, at UNRRA administrative offices, by military authorities’ or ‘by private employers’.^82^ Relief worker Duchesne-Cripps wrote that ‘it was realized early by UNRRA that teams recruited abroad for Germany might usefully be supplemented by certain especially able persons in the various categories’.^83^ While important literature on DP labour in refugee camps exists,^84^ there is hardly any research on DPs as part of the staff of UNRRA or the IRO.^85^

Towards the end of their mandate, UNRRA officials increasingly discussed the replacement of so-called Class I personnel with Class II personnel: ‘Class I personnel will be replaced by Class II employees [sic] and other qualified workers “drawn from among displaced persons” where possible’.^86^ As Salvatici has emphasized, ‘budget constraints forced the organization [UNRRA] to reduce the international staff (Class I), and to rely more extensively on the recruitment from inhabitants within the Assembly Centres (Class II)’.^87^ By ‘recruiting “Class II” employes [sic] from the ranks of DPs cared for in the UNRRA-administered centers in the three western zones’, it was possible to keep ‘a balance of one UNRRA employe [sic] per 150 displaced persons’.^88^ Thus, the increase in Class II staff made it possible for the organization to continue its mission despite its decreasing budget. The reduction in the number of personnel involved in the DP operation in Germany was connected to UNRRA's preparation for the taking over of responsibility by the IRO. The goal of the new employment policy was ‘to create an organization in size and form suitable for assumption of control by IRO’.^89^ When the IRO was taking over UNRRA's DP operations,

[t]he Report of the Committee on Finance of the IRO has recommended a reduction in the number of imported personnel now being used in the Displaced [Persons] programmes on the assumption that ‘many of the functions now being performed by regular UNRRA staff’ may ‘be performed by Displaced Persons themselves’.

According to the working papers of the Joint UNRRA–UN Planning Commission, the IRO intended to work more closely with the DPs than UNRRA had done. While it is clear that UNRRA, and even more so the IRO, relied on the work of the DP Class II employees, this was not reflected in their status within the organization. Only a few of the DPs recruited by UNRRA or the IRO were employed as Class I personnel,^90^ which was mainly reserved for non-DP workers.^91^ These differences in employment had an effect on the labour conditions of these DPs since Class I status would have meant higher wages and a higher status within the UN system: ‘salaries, promotions, leave and benefits were very different for the two “classes”, a fact which emphasized their asymmetrical status’.^92^ Besides the wage gap, Class II DP workers could ‘not participate in the bonus or compensation schemes’ for Class I employees. Furthermore, employees from Class II ‘in general filled the lower grade posts and posts to which no grade is assigned, such as those for liftmen, doorkeepers, etc.’^93^ Salvatici also points out how they had ‘limited access to military stores’, which ‘represented a boundary line which divided DPs recruited locally from the international staff’.^94^ The different status of DP staff was further aggravated by the fact that the non-DP staff, as Salvatici argues, ‘continued to see them mainly as displaced persons and not as colleagues’.^95^ DPs themselves ‘objected to the term “Class II”’,^96^ which did not correspond to their responsibilities, which were the same or similar to those of Class I personnel.

Regardless of the specific type of employment DPs held – whether they were officially employed by UNRRA or the IRO or not – their significance to the work of international relief teams is evident in sources by non-DP relief workers. UNRRA relief worker Kathryn Hulme, for example, recounted that, after having been instructed about the residents and infrastructure of the Wildflecken camp, they

picked up interpreters, […] cars to transport us, and Polish drivers to piece these together every time they fell apart. We picked up camp leaders, the police chiefs and the priesthood […] all the people on whom we had to depend.^97^

Besides economic considerations, one reason why relief workers depended on DPs and why they strived to collaborate with them was the former's lack of knowledge about the daily needs and challenges that existed among the diverse groups within the refugee population, as well as their lack of certain language skills. One of the arguments pursued in this paper is that it was exactly this knowledge and expertise that allowed DP workers to exercise proxy agency for their fellow DPs. In the relief workers’ reports, the vital role that language played in effectively administering relief work is mentioned multiple times. Possessing the required language skills could therefore be seen as a key indicator of agency in the context of the postwar administration of care and support to displaced people. And it was this ability that international, non-DP relief workers very often lacked. Hulme pointed out that in the team with which she first worked in Germany, there were people with knowledge of ‘French, Dutch, Norwegian, Flemish and English’.^98^ Knowledge of Eastern European languages, or of German – which many DPs spoke or at least partly understood^99^ – was not mentioned. Duchesne-Cripps also underlined these ‘general limitations on personal usefulness [of relief workers] through lack of knowledge of a language comprehensible to the people’.^100^

In her book, McNeill wrote that the first phase of her team's work with the DPs in Europe was difficult because ‘we knew so little, so pitifully little, about the work ahead of us. […] We were ready and anxious to heal. But did we really understand the nature of the disease?’^101^ Duchesne-Cripps also noted that DP workers could better empathize with the DPs’ persecution and displacement experiences:

Especially in welfare work, a worker of the same nationality as the Displaced Persons themselves was often better than a foreign worker. In several instances in the teams, workers of special ability for welfare work were found amongst the [displaced] people. Understanding of the needs of the people and experience of the actual conditions in which they lived were in such cases of greater value than a social work training, which indeed they did not possess. It was with such help that welfare in camps would be worked out.^102^

Closeness to the DP experience, as well as language skills, were thus some of the main advantages of employing and cooperating with DPs.

UNRRA did, however, also encounter challenges in its work with DPs. These challenges provide a clear indication of the kind of refugee agency that could result from the cooperation and employment of DPs as UNRRA staff. This becomes especially evident regarding the sensitive issue of repatriation. The general stance of UNRRA has been ‘to encourage maximum employment of displaced persons, so long as such employment does not interfere with repatriation’^103^ – meaning the return of DPs to their places of origin. The possibility that DPs employed or working for UNRRA could use their position within the UNRRA system to contribute to ‘anti-repatriation activities’ was therefore seen as a problem for which ‘close supervision and remedial action’ was suggested.^104^ The use of Class II DP personnel in fields of operation connected to repatriation was seen as disadvantageous:

No great degree of effectiveness in connection with repatriation can be expected from Class II employees whose lack of an objective attitude toward their own problems is evidenced by their opposition to repatriation.^105^

The same scepticism was also directed at DP representatives who were opposed to repatriation and might share this disapproval with other DPs: ‘UNRRA staff must immediately discontinue working relationships with improperly accredited national representatives and have them removed from camps’. It was deemed necessary by UNRRA to check ‘“the self-government” machinery in each camp to ensure that it is conducive to repatriation and that individuals or groups of displaced persons responsible are not actively deterring repatriation’.^106^ UNRRA's decision not to involve DPs in repatriation matters offers intriguing insights into the type of agency it was feared they could exercise through participation in these fields of operation.

With regard to Class II employees, it was also argued that they could not be placed in ‘certain positions, e.g., those of distribution observers, Public Relation Officers […] because it is essential that they be entirely free from local pressure or else that they are trained in the main contributing countries’.^107^ These examples show that for certain sensitive issues and certain fields of work, DPs were not considered ‘neutral’ enough by UNRRA, who feared that they could exercise too much influence upon their fellow DPs. The agency that could result from the incorporation of Class II staff in repatriation activities was viewed as a potential risk to UNRRA's mission. What can be observed here is that UNRRA officials feared that DP employees would undermine the main purpose of UNRRA's policy – preparing DPs for repatriation – from *within*, when given the chance. So even though there is no direct statement of the refugee agency in the above-cited passages, what becomes apparent is that UNRRA was suspicious of the DP workers’ potential ability to influence how their fellow DPs felt about repatriation and therefore decided not to recruit them for positions where they could exercise this agency.

This paper has argued that by working in their own workshops as well as through their collaboration with non-DP UNRRA officials, refugee workers could exercise both personal and proxy agency. This became particularly evident in the examples of DP doctors, DP leaders and DP translators described in this paper. Due to the absence of personal testimonies from Class II employees, it remains challenging to determine how DPs employed as part of UNRRA's or the IRO's staff felt about working for the organizations. The potential future availability of UNRRA and IRO personnel files might offer further understanding of this matter.

While the vocational training for DPs and UNRRA's involvement in providing relief and rehabilitation for refugees have been addressed in many works on postwar refugee camps, as well as in works focusing on the labour requirements for DPs, not enough attention has been paid to the agency that could result from the DPs’ collaboration in relief and rehabilitation work, both for the DPs themselves as well as the non-DP relief officers.

The aim of this paper was therefore twofold. Firstly, it sought to demonstrate what forms of refugee agency could emerge from the cooperation of DPs with non-DP relief personnel by offering examples of personal and proxy agency among refugees working inside the camps, or as part of UNRRA's DPs operations. Secondly, it aimed to provide new insights into how this collaboration benefited the work of the UN institutions and thereby the administration of relief and rehabilitation in general.

The paper has demonstrated that, to a certain extent, refugee workers could use their skills and positions to improve their own livelihoods. This could be achieved by securing additional rations or wages, or by gaining valuable information about the resettlement process, as Stanislawa's experience illustrates. Through their involvement in relief work, DPs were also able to improve the circumstances of their fellow DPs, as the cases of the DP doctors and the Polish DP translator demonstrate, and thereby exercise certain elements of proxy agency.

Furthermore, the article has shown that non-DP relief workers and UNRRA officials considered it very important and beneficial to closely cooperate with DPs for various reasons. DPs were deliberately employed by UNRRA as Class II personnel to fulfil the organization's mandate more effectively and to save money on more expensive international staff. At the same time, however, the organization feared that DPs might undermine its official policies, such as those related to repatriation.

While many DPs used their skills in their private workshops or for work outside the camp, others were employed by UNRRA or the IRO and worked as part of their staff, despite their lower status within the organizational hierarchy. The DPs’ knowledge of certain languages and their closeness to their fellow DPs’ persecution and displacement experiences, as well as UNRRA's need to curtail the employment of Class I personnel towards the end of its mandate, all led to DPs being sought-after workers for administering relief and rehabilitation in postwar Europe. This paper has demonstrated that the agency of refugee workers and non-DP relief workers was, in fact, closely interrelated. It showed how international relief teams depended heavily on the cooperation of DPs in their relief and rehabilitation efforts, while also illustrating that this involvement could, in turn, strengthen the agency of the DPs themselves.

## Notes

1. S. Gemie, L. Humbert, F. Reid and L. Ingram, *Outcast Europe: Refugees and Relief Workers in an Era of Total War 1936–48* (London 2012), 193.

2. See also the use of the term ‘refugee-workers’, in D. Kay and R. Miles, ‘Refugees or Migrant Workers: The Case of the European Volunteer in Britain (1946–1951)’, *Journal of Refugee Studies*, 1, 3 and 4 (1988), 214–36, 215. Contrary to its use in this paper, however, Kay and Miles used the term to specifically refer to those DPs who emigrated through labour recruitment programmes, such as the ‘European Voluntary Workers’ programme, to the United Kingdom.

3. For more information on the labour policies of the Allies, see S. Salvatici, ‘From Displaced Persons to Labourers: Allied Employment Policies in Post-War West Germany’, in J. Reinisch and E. White (eds) *The Disentanglement of Populations: Migration, Expulsion and Displacement in Post-War Europe, 1944–9* (London 2011), 210–28. For more information on the UNRRA university for displaced persons, see A. Holian, ‘Displacement and the Post-War Reconstruction of Education: Displaced Persons at the UNRRA University of Munich, 1945–1948’, *Contemporary European History*, 17, 2 (May 2008), 167–95. For more information on the vocational schools, for example, in Landsberg, see A. Schein, ‘She'erit ha-Peletah: Women in DP Camps in Germany’, *Shalvi/Hyman Encyclopedia of Jewish Women*, 31 December 1999, Jewish Women's Archive, https://jwa.org/encyclopedia/article/sheerit-ha-peletah-women-in-dp-camps-in-germany (accessed 4 January 2023).

4. See J. Reinisch, ‘Refugees and Labour in the Soviet Zone of Germany, 1945–9’, in J. Reinisch and E. White (eds) *The Disentanglement of Populations: Migration, Expulsion and Displacement in Post-War Europe, 1944–9* (London 2011), 185–209, here 185.

5. See J. Lingelbach, ‘Chapter 2: The Postwar Refugee Regime and the Imperial Order of Things’, in *On the Edges of Whiteness* (Oxford 2020), 65–103, 68.

6. See S. Salvatici, ‘From Displaced Persons to Labourers: Allied Employment Policies in Post-War West Germany’, in J. Reinisch and E. White (eds) *The Disentanglement of Populations: Migration, Expulsion and Displacement in Post-War Europe, 1944–9* (London 2011), 210–28, or R. Blumenthal, ‘Rehabilitation Through Labour: Welfare or Control in a Postwar Austrian Internment Camp’, in G. Anderl, L. Erker and C. Reinprecht (eds) *Internment Refugee Camps* (Bielefeld 2022), 59–72.

7. G. H. Elder Jr., M. Kirkpatrick Johnson and R. Crosnoe, ‘The Emergence and Development of Life Course Theory’, in J. T. Mortimer and M. J. Shanahan (eds) *Handbook of the Life Course* (Boston, MA 2003), 3–19, 14.

8. A. Bandura, ‘Social Cognitive Theory: An Agentic Perspective’, *Annual Review of Psychology*, 52, 1 (2001), 1–26. 13.

9. M. Garcia-Godinez, ‘Institutional Proxy Agency: A We-Mode Approach’, in M. Garcia-Godinez and R. Mellin (eds) *Tuomela on Sociality* (Cham 2023), 151–76, 151.

10. Germany – Arolsen HQ – Employment of D. Ps (645), S-1278-0000-0196-00001, United Nations Archives and Records Management Section, New York (UNA), 2.

11. S. Salvatici, ‘“Help the People to Help Themselves”: UNRRA Relief Workers and European Displaced Persons’, *Journal of Refugee Studies*, 25, 3 (2012), 428–51, 440.

12. Ibid, 440–1.

13. See Salvatici, ‘Help the People to Help Themselves’, 444–5 and N. Aksamit, ‘Paving the Road to Reconciliation: The Training and Practice of the Friends Relief Service in Post-War Reconciliation, 1943 to 1947’, *European Review of History: Revue européenne d’histoire*, 30, 6 (2023), 861–87, 868–9.

14. Gemie et al., *Outcast Europe*, 147.

15. See A. Duchesne-Cripps, *The Mental Outlook of the Displaced Persons as Seen Through Welfare Work in Displaced Persons Camps* (Cambridge 1955), 3–5.

16. See Private Papers of T. S. Hall, 14461, Imperial War Museum (IWM), London.

17. Christine Schmidt and Dan Stone highlight several published memoirs by female UNRRA relief workers in their paper ‘Women in Child Search: A Gendered View of Post-World War II Reconstruction’, *European Review of History: Revue européenne d’histoire*, 30, 6 (2023), 888–906, 890, 901.

18. See M. McNeill, *By the Rivers of Babylon: A Story of Relief Work among the Displaced People of Europe* (London 1950), 9.

19. K. Hulme, *The Wild Place* (Kindle Edition Plunkett Lake Press 2017/1st edition 1953).

20. Bandura, ‘Social Cognitive Theory: An Agentic Perspective’, 13.

21. R. Bailey, ‘Disease, DPs, and DDT: A Global Health Perspective on the History of Refugee Relief’, *Itinerario*, 46, 2 (2022), 233–50, 245.

22. For additional insights into the complex and sometimes strained relationship between UNRRA and the military authorities, see, for example, Salvatici, ‘From Displaced Persons to Labourers’, 219.

23. See Blumenthal, ‘Rehabilitation through Labour’, 60.

24. L. Humbert, ‘French Politics of Relief and International Aid: France, UNRRA and the Rescue of European Displaced Persons in Postwar Germany, 1945–47’, *Journal of Contemporary History*, 51, 3 (2016), 606–34, 631.

25. Humbert, ‘French Politics of Relief and International Aid’, 629.

26. Blumenthal, ‘Rehabilitation through Labour’, 59.

27. Ibid, 63–4.

28. Salvatici, ‘From Displaced Persons to Labourers’, 219–20.

29. Blumenthal, ‘Rehabilitation through Labour’, 70.

30. Ibid, 64.

31. Salvatici, ‘From Displaced Persons to Labourers’, 211.

32. Hulme, *Wild Place*, chapter 12, paragraph 32.

33. ‘Repatriation and Employment Prime Aims of UNRRA in British Zone during 1946’, 8 February 1947, S-1253-0000-0417-00001, UNA, 19.

34. Salvatici, ‘From Displaced Persons to Labourers’, 213.

35. Ibid, 215.

36. ‘Repatriation and Employment Prime Aims of UNRRA in British Zone during 1946’, 8 February 1947, S-1253-0000-0417-00001, UNA, 19.

37. Notable exceptions here are the paper by M. M. Feinstein, ‘Jewish Women Survivors in the Displaced Persons Camps of Occupied Germany’, *Shofar*, 24, 4 (Summer 2006) and the last section of Salvatici's paper ‘From Displaced Persons to Labourers’, 220–3.

38. See Feinstein, ‘Jewish Women Survivors in the Displaced Persons Camps of Occupied Germany’, 81, 83.

39. Ibid, 88.

40. Blumenthal, ‘Rehabilitation Through Labour’, 70.

41. See Margarete Myers Feinstein, ‘Jewish Women Survivors in the Displaced Persons Camps of Occupied Germany: Transmitters of the Past, Caretakers of the Present, and Builders of the Future’, *Shofar: An Interdisciplinary Journal of Jewish Studies* 24, 4 (2006), 83–4, https://doi.org/10.1353/sho.2006.0090.

42. Feinstein, ‘Jewish Women Survivors in the Displaced Persons Camps of Occupied Germany’, 70.

43. DP women were in especially high demand in the health sector as ‘the rise in the number of inmates in the DP camps led to an ongoing shortage of nurses’, in: Schein, ‘She'erit ha-Peletah: Women in DP Camps in Germany’.

44. A. Grossmann, *Jews, Germans, and Allies. Close Encounters in Occupied Germany* (Princeton 2007), 214.

45. Feinstein, ‘Jewish Women Survivors in the Displaced Persons Camps of Occupied Germany’, 85.

46. Ibid.

47. Hulme, *Wild Place*, chapter 5, paragraph 61.

48. Blumenthal, ‘Rehabilitation Through Labour’, 66.

49. Ibid, 70.

50. Feinstein, ‘Jewish Women Survivors in the Displaced Persons Camps of Occupied Germany’, 83–5.

51. Cited from an interview conducted by Feinstein with Hilde Mantelmacher, in Feinstein ‘Jewish Women Survivors in the Displaced Persons Camps of Occupied Germany’, 83–4.

52. See Feinstein, ‘Jewish Women Survivors in the Displaced Persons Camps of Occupied Germany’, 83–4.

53. Hulme, *Wild Place*, chapter 7, paragraph 32.

54. Hulme, *Wild Place*, chapter 14, paragraph 9.

55. Ibid, paragraph 10.

56. Hulme, *Wild Place*, chapter 15, paragraph 32.

57. Ibid, paragraph 39.

58. Hulme describes the Polish Countess as a very educated woman, speaking several languages, see: Hulme, *Wild Place*, chapter 2, paragraph 26.

59. Hulme, *Wild Place*, chapter 2, paragraphs 42 and 44.

60. Hulme, *Wild Place*, chapter 5, paragraphs 71 and 80.

61. Ibid, Paragraph 83.

62. J. Reinisch, ‘Old Wine in New Bottles? UNRRA and the Mid-Century World of Refugees’, in M. Frank and J. Reinisch (eds) *Refugees in Europe, 1919–1959* (London 2017), 147–76, 161.

63. A. Holian, *Between National Socialism and Soviet Communism: Displaced Persons in Postwar Germany. Social History, Popular Culture, and Politics in Germany* (Ann Arbor 2011) 48.

64. Ibid, 48–9.

65. Private Papers of T. S. Hall, 6 December 1945, IWM London.

66. Duchesne-Cripps, *Mental Outlook*, 25, 26.

67. Cohen, ‘Between Relief and Politics’, 442.

68. Private Papers of T. S. Hall, 14461, 29 November 1945; 16 October 1945; 30 November 1945, IWM London.

69. McNeill, *Rivers of Babylon*, 42.

70. K. Nowak, ‘A Gloomy Carnival of Freedom Sex, Gender, and Emotions among Polish Displaced Persons in the Aftermath of World War II’, *Aspasia*, 13, 1 (2019), 113–34, 115.

71. Ibid, 114, 130.

72. See Žakelj, *Life in the Refugee Camps, July–December 194*5, 6 July 1945; 4 November 1945, http://bbhhs96.dyndns.org/~zakeljdiary/1945life.htm (accessed 30 November 2025).

73. See Salvatici, ‘Help the People to Help Themselves’, 443.

74. ‘Joint UNRRA – UN Planning Commission, Working Paper’, August 1946, S-1252-0000-0074, UNA, 26.

75. See Monthly Statistical Report on Displaced Persons Operations, Germany, March 1947, S-1261-0000-0005-00001, UNA, 177.

76. Duchesne-Cripps, *Mental Outlook*, 25.

77. See G. D. Cohen, ‘Between Relief and Politics: Refugee Humanitarianism in Occupied Germany 1945–1946’, *Journal of Contemporary History*, 43, 3 (2008), 437–49, 440.

78. See UNRRA London, 23 January 1947, S-1304-0000-0122-00001, UNA, 107.

79. Salvatici, ‘Help the People to Help Themselves’, 444.

80. Duchesne-Cripps, *Mental Outlook*, 23.

81. Salvatici, ‘Help the People to Help Themselves’, 436.

82. ‘Note on Employment of Displaced Persons, No. 118’, S-1252-0000-0079-00001, UNA, 218.

83. Duchense-Cripps, *Mental Outlook*, 25.

84. See Blumenthal, ‘Rehabilitation through Labour’ and Salvatici, ‘From Displaced Persons to Labourers’.

85. A notable exception here is Salvatici's reference to UNRRA Class II employees in her article on relief and rehabilitation work, ‘Help the People to Help Themselves’.

86. ‘Personnel Strength Reduction to 2,990 Scheduled for UNRRA's DP Operations’, 15 September 1946, S-1253-0000-0417-00002, UNA, 43.

87. Salvatici, ‘Help the People to Help Themselves’, 443.

88. ‘Keep Balance by Recruitment of Class II DPs’, 8 February 1947, S-1253-0000-0417-00001, UNA, 18.

89. ‘Repatriation is Primary Duty of All Remaining Employes’, 15 September 1946, S-1253-0000-0417-00002, UNA, 36.

90. See ‘Joint UNRRA-UN Planning Commission, Working Paper’, 30 August 1946, S-1252-0000-0074, UNA, 26.

91. See Salvatici, ‘Help the People to Help Themselves’, 444.

92. Ibid.

93. ‘Note on Class II Employees’, 27 January 1947, S-1252-0000-0079-00001, UNA, 111.

94. Salvatici, ‘Help the People to Help Themselves’, 444.

95. Ibid.

96. Grimon, Director of Personnel, ERO to CHQ Arolsen, 17 June 1946, S-1249-0000-0074-00001, UNA, 15.

97. Hulme, *Wild Place*, chapter 2, paragraph 20.

98. Hulme, *The Wild Place*, chapter 1, paragraph 2.

99. See Duchesne-Cripps, *Mental Outlook*, 24.

100. Duchesne-Cripps, *Mental Outlook*, 24.

101. McNeill, *Rivers of Babylon*, 15.

102. Duchesne-Cripps, *Mental Outlook*, 25.

103. ‘Note on Employment of Displaced Persons, No. 118’, 27 January 1947, S-1252-0000-0079-00001, UNA, 218.

104. ‘Re-organization of UNRRA DP operation in Germany, UNRRA Headquarters Directive’, 3 July 1946, S-1261-0000-0005-00001, UNA, 189.

105. ‘Chapter IV. Displaced Persons Operations. Development of UNRRA Displaced Persons Program’, 1947, S-1252-0000-0278-00001, UNA, 163.

106. Displaced Persons Division, ERO, 21 January 1947, S-1252-0000-0079-00001, UNA, 201.

107. ‘Note on Class II Employees’, 27 January 1947, S-1252-0000-0079-00001, UNA, 111.

